# Disrupted glucose homeostasis and skeletal-muscle-specific glucose uptake in an exocyst knockout mouse model

**DOI:** 10.1016/j.jbc.2021.100482

**Published:** 2021-02-27

**Authors:** Brent A. Fujimoto, Madison Young, Nicole Nakamura, Herena Ha, Lamar Carter, Matthew W. Pitts, Daniel Torres, Hye-Lim Noh, Sujin Suk, Jason K. Kim, Noemi Polgar

**Affiliations:** 1 Department of Anatomy, Biochemistry, and Physiology, John A. Burns School of Medicine, University of Hawaii, Honolulu, Hawaii, USA; 2 Department of Cell and Molecular Biology, John A. Burns School of Medicine, University of Hawaii, Honolulu, Hawaii, USA; 3 Program in Molecular Medicine, University of Massachusetts Medical School, Worcester, Massachusetts, USA; 4 Division of Endocrinology, Metabolism, and Diabetes, Department of Medicine, University of Massachusetts Medical School, Worcester, Massachusetts, USA

**Keywords:** diabetes, exocyst complex, glucose transporter, insulin resistance, skeletal muscle, EE, energy expenditure, GLUT4, glucose transporter type 4, GSV, GLUT4 storage vesicle, GTT, glucose tolerance test, HFD, high-fat diet, HGP, hepatic glucose production, HSA-MCM, human skeletal muscle actin Mer-Cre-Mer, IP, intraperitoneal, IRS-1, insulin receptor substrate 1, ITT, insulin tolerance test, Pdk1, protein-dependent kinase 1, RER, respiratory exchange ratio, T2D, type 2 diabetes

## Abstract

Skeletal muscle is responsible for the majority of glucose disposal following meals, and this is achieved by insulin-mediated trafficking of glucose transporter type 4 (GLUT4) to the cell membrane. The eight-protein exocyst trafficking complex facilitates targeted docking of membrane-bound vesicles, a process underlying the regulated delivery of fuel transporters. We previously demonstrated the role of exocyst subunit EXOC5 in insulin-stimulated GLUT4 exocytosis and glucose uptake in cultured rat skeletal myoblasts. However, the *in vivo* role of EXOC5 in skeletal muscle remains unclear. Using mice with inducible, skeletal-muscle-specific knockout of exocyst subunit EXOC5 (*Exoc5*-SMKO), we examined how muscle-specific disruption of the exocyst would affect glucose homeostasis *in vivo*. We found that both male and female *Exoc5*-SMKO mice displayed elevated fasting glucose levels. Additionally, male *Exoc5*-SMKO mice had impaired glucose tolerance and lower serum insulin levels. Using indirect calorimetry, we observed that male *Exoc5*-SMKO mice have a reduced respiratory exchange ratio during the light period and lower energy expenditure. Using the hyperinsulinemic–euglycemic clamp method, we further showed that insulin-stimulated skeletal muscle glucose uptake is reduced in *Exoc5*-SMKO males compared with wild-type controls. Overall, our findings indicate that EXOC5 and the exocyst are necessary for insulin-stimulated glucose uptake in skeletal muscle and regulate glucose homeostasis *in vivo*.

Skeletal muscle is responsible for 80 to 90% of insulin-stimulated glucose uptake in the body (1). Insulin triggers the targeted trafficking of GLUT4 to the cell membrane, enabling glucose uptake into cells, and insulin-resistant cells show defects in GLUT4 trafficking to the membrane (2). The “solute carrier 2A” (SLC2A or GLUT) family of glucose transporters allow glucose to cross the cell membrane by facilitated diffusion. GLUT1 is constitutively present on the plasma membrane of cells in most tissues, while GLUT4 is the principal insulin responsive glucose transporter (3). In basal state, GLUT4 is sequestered in GLUT4 storage vesicles (GSVs) in the cytosol (4). When stimulated by insulin, GSVs are translocated along the cytoskeleton toward the cell membrane (5), where they are “docked” to the plasma membrane by tethering protein complexes (6). It is only after docking, when the membrane of the GSVs can be fused to the plasma membrane with the assistance of SNAP Receptor (SNARE) proteins (7).

In 3T3-L1 cells, it has been demonstrated that the exocyst protein complex is essential for the delivery of GLUT4-containing vesicles to the plasma membrane in response to insulin (8). The exocyst complex is an eight-member holocomplex that ensures that certain exocytic vesicles are trafficked to the appropriate location on the plasma membrane (9). The exocyst complex member EXOC6 is necessary for GLUT4 exocytosis in adipocytes (10), and the overexpression of EXOC3 and EXOC4 increases the magnitude of insulin-stimulated glucose uptake in cultured adipocytes (11).

The vast majority of the previous research demonstrating the essentiality of the exocyst complex for insulin-stimulated GLUT4 exocytosis has been performed *in vitro* in cultured adipocytes (12, 13). We have recently demonstrated that the exocyst complex is necessary for GLUT4 exocytosis in cultured skeletal myoblasts and myotubes as well (14). In healthy individuals, skeletal muscle takes up 80 to 90% of glucose in response to insulin, and one of the earliest detectable signs of insulin resistance preceding type 2 diabetes (T2D) is reduced insulin-stimulated glucose uptake in skeletal muscle (1). In the skeletal muscle cells of people with T2D, the insulin signaling pathway and subsequent GLUT4 exocytosis are suppressed (15), impairing glucose uptake into cells. A detailed map of the processes between the activation of the insulin receptor and the exocytosis of GLUT4 in skeletal muscle is yet to be completed. The discovery of new effectors of the insulin signaling pathway could provide novel therapeutic approaches for the treatment of insulin resistance and T2D (16). In this article, we are the first to report that *in vivo* disruption of the exocyst complex affects skeletal muscle glucose uptake and homeostasis in a novel conditional knockout mouse model.

## Results

### Generation of skeletal-muscle-specific *Exoc5* knockout mice

**Figure 1:**
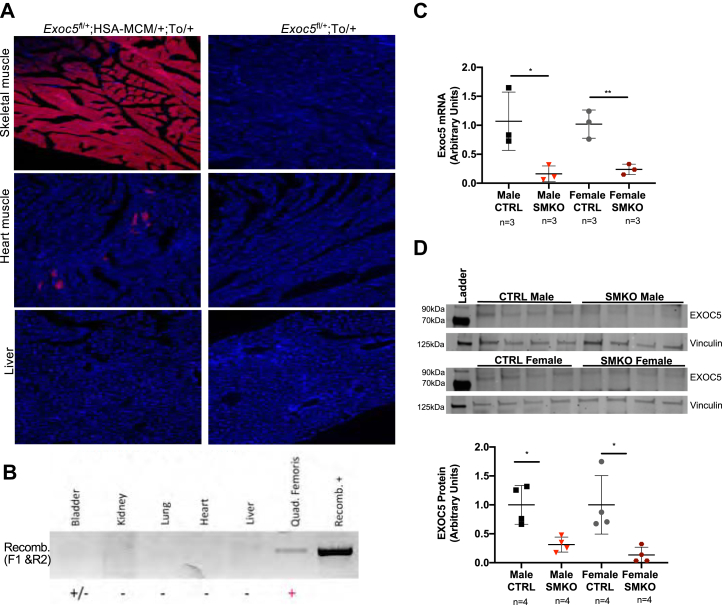
**Conditional knockouts of *Exoc5* in mouse skeletal muscle were generated.***A*, fluorescent microscopy evaluating tissue-specific Cre expression using a mouse line with a tdTomato (To) reporter allele. The tissue sections are from female mice. *B*, recombination PCRs from genomic DNA of various tissues of tamoxifen-treated male mice to detect deletion of exons 7 to 10. *C*, real-time quantitative PCR measuring skeletal-muscle-specific EXOC5 mRNA expression (normalized to RPLP0) in *Exoc5*-SMKO and CTRL mice. *D*, representative image of a Western blot analysis (*upper panel*) used for the quantification (*lower panel*) of EXOC5 protein levels (normalized to Vinculin) in the soleus of *Exoc5*-SMKO and CTRL mice. *Error bars* = standard deviation, ∗*p* ≤ 0.05, ∗∗*p* ≤ 0.01.

Previously, we demonstrated that the exocyst subunit EXOC5 is essential for insulin-stimulated GLUT4 exocytosis and glucose uptake in cultured rat skeletal myoblasts (17). To study the consequences of *in vivo* disruption of *Exoc5* and the exocyst, we generated skeletal-muscle-specific *Exoc5* conditional knockout (*Exoc5*-SMKO) mice. We crossed our “floxed” *Exoc5* mouse strain with the human skeletal muscle actin Mer-Cre-Mer (HSA-MCM) mice strain and treated them with tamoxifen to induce Cre recombinase activation and thus inactivate *Exoc5* in a skeletal-muscle-specific manner (Fig. 1*A*).

We utilized a *tdTomato* reporter mouse strain to confirm skeletal-muscle-specific Cre activity. As anticipated, tamoxifen-treated Exoc5^fl/+^;HSA-MCM/+;To/+ female mice expressed tdTomato almost exclusively in skeletal muscle tissues (Fig. 1*A*, *top panel*). A few cardiomyocytes also expressed tdTomato (Fig. 1*A*, *middle panel*), but no tdTomato expression was detected in the liver (Fig. 1*A*, *lower panel*), lung, or kidney tissues (*data not shown*). We designed a PCR-based assay to test genomic DNA recombination, where a product is obtained only when exons 7 to 10 are removed from the *Exoc5* gene. Recombination of genomic DNA was detected in male *Exoc5*-SMKO mice in skeletal muscle and bladder samples, but not in other tissues assayed (Fig. 1*B*).

Quantitative real-time PCR revealed that the knockout muscle tissue has significantly less *Exoc5* mRNA expressed than controls (Fig. 1*C*) in both sexes. Western blot analysis demonstrated that *Exoc5*-SMKO male and female mice have decreased EXOC5 protein levels in soleus muscle tissue compared with littermate controls (Fig. 1*D*). Thus, in *Exoc5*-SMKO mice, following tamoxifen treatment, the Cre-recombinase was expressed in a skeletal-muscle-specific manner, and EXOC5 expression was markedly diminished in skeletal muscle tissue at both the RNA and protein levels, with similar efficiency in both male and female mice. Lastly, skeletal-muscle-specific ablation of *Exoc5* did not impair neuromuscular muscle function in either sex, as *Exoc5*-SMKO performed similarly to control mice in open field and rotarod testing (Fig. S1).

### Altered energy expenditure and respiratory exchange ratio in *Exoc5*-SMKO mice

**Figure 2:**
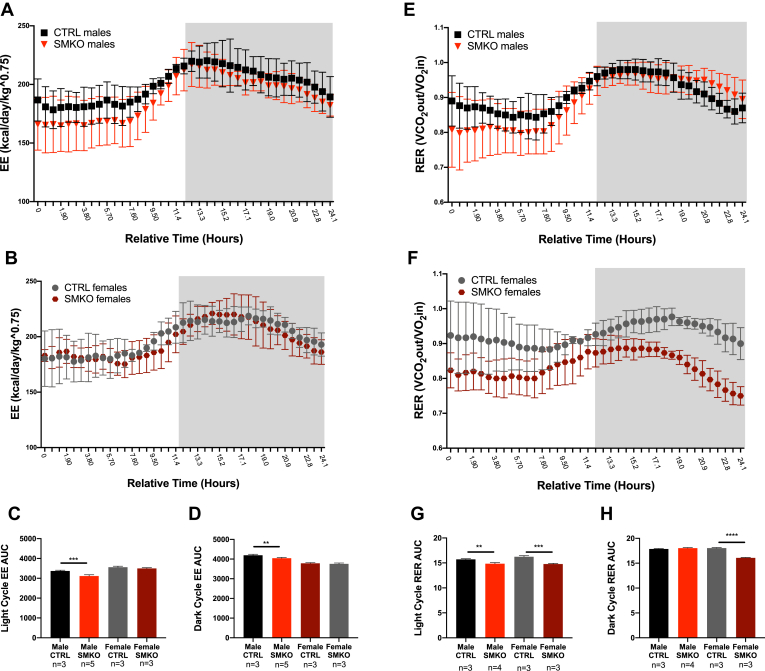
**Indirect calorimetric analysis indicates a shift from glucose utilization in skeletal muscle specific *Exoc5* knockout mice during daylight hours and a lower energy expenditure.** Indirect calorimetry was performed on *Exoc5*-SMKO and CTRL mice by placing them in metabolic cages. Energy expenditure (EE) for male (*A*) and female (*B*) mice was calculated over a 24-h period, the dark cycle is symbolized by the *grayed-out area*. Area under the curve was calculated for the light period (*C*) and the dark period separately (*D*). The respiratory exchange ratio (RER) for male (*E*) and female (*F*) mice was calculated based on the ratio of O_2_ flowing into the cage and the CO_2_ flowing out of the cage over a 24-h period, the dark cycle is symbolized by the *grayed-out area*. Area under the curve was calculated for the light period (*G*) and the dark period separately (*H*). *Error bars* = standard deviation. ∗*p* ≤ 0.05, ∗∗*p* ≤ 0.01, ∗∗∗*p* ≤ 0.001, ∗∗∗∗*p* ≤ 0.0001.

An indirect calorimetry study was conducted to assess whole-body energy balance in *Exoc5*-SMKO mice. The energy expenditure (EE) rate over a 24-h period was significantly reduced during both light and dark cycles in male *Exoc5-*SMKO mice, but not in female mice (Fig. 2, *A*–*D*). Additionally, respiratory exchange ratio (RER), measured over the course of a 24-h period, was significantly lower in male *Exoc5*-SMKO mice during light cycle and in female *Exoc5*-SMKO mice during both the light and dark cycle suggesting decreased glucose utilization in these mice compared with controls (Fig. 2, *E*–*H*). There were no significant differences in body weight, activity level (Fig. S2, *A*–*C*), and food or water intake (data not shown) between *Exoc5*-SMKO and control mice. These observations suggest that skeletal-muscle-specific disruption of *Exoc5* affects whole-body energy substrate utilization.

### Altered glucose homeostasis in *Exoc5*-SMKO mice

**Figure 3:**
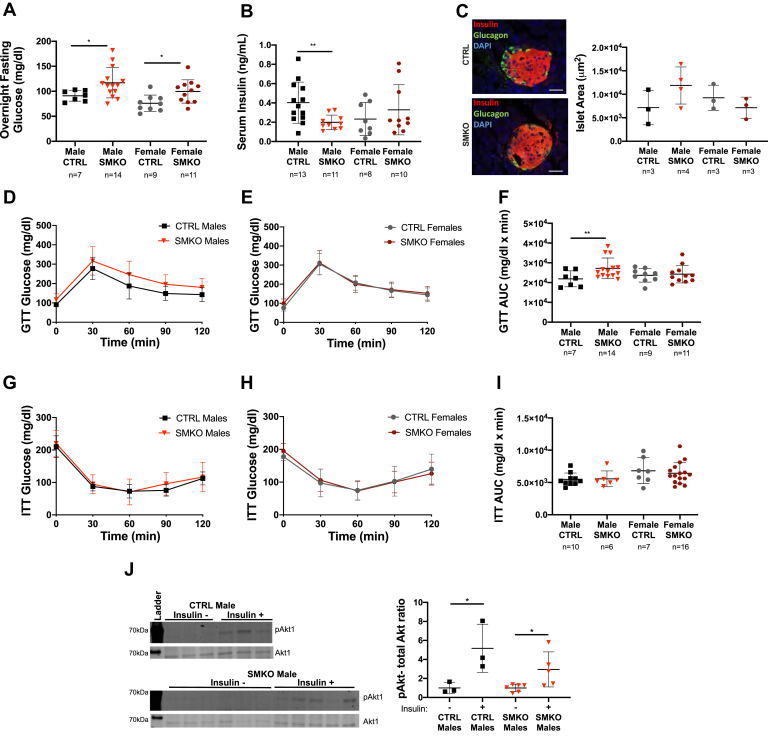
**Glucose homeostasis is perturbed in *Exoc5* skeletal muscle knockout animals.***A*, *Exoc5*-SMKO and CTRL animals were fasted overnight, and fasting blood glucose concentrations were measured *via* tail bleed. *B*, six-hour fasting insulin levels were measured in *Exoc5*-SMKO and control littermate animals using serum samples and an ELISA assay. *C*, representative images of pancreatic islet immunofluorescent staining (*left panel*) and quantification of islet size in *Exoc5*-SMKO and CTRL mice (*right panel*). White scale bars, 20 μm. *D* and *E*, for the glucose tolerance testing, blood glucose concentrations were measured at 30-min intervals following an intraperitoneal bolus of glucose (1 mg/kg). *F*, quantification of area under the curve for data shown in *D* and *E*. *G* and *H*, prior to insulin tolerance testing, 4- to 5-month-old *Exoc5*-SMKO and CTRL mice were fasted for 6 h and blood glucose levels were measured by tail bleed. Following fasting glucose measurement, an intraperitoneal injection of insulin was administered (1 U/kg). Blood glucose was measured at baseline and every 30 min for 2 h by tail blood sampling. *I*, area under the curve calculations for data presented in *G* and *H*. *J*, Western blots (*left panel*) and quantification (*right panel*) of insulin signaling pathway component Akt in *Exoc5*-SMKO and CTRL following insulin injection. *Error bars* = standard deviation, ∗*p* ≤ 0.05; ∗∗*p* ≤ 0.01.

In order to analyze the effect of the *Exoc5* knockout in skeletal muscle on glucose homeostasis, we first measured fasting glucose and insulin levels. Blood glucose levels following overnight fast in male and female *Exoc5*-SMKO mice were significantly higher than in littermate controls (Fig. 3*A*), suggesting impaired glucose homeostasis and hepatic insulin resistance.

We also measured serum insulin levels by ELISA following a 6-h fast and found that male *Exoc5*-SMKO mice had significantly lower insulin levels than controls (Fig. 3*B*). This effect was selective to male mice because female *Exoc5*-SMKO mice had similar insulin levels to their littermate controls. The examination of pancreatic islets found no significant differences in islet size compared with controls in either sex (Fig. 3*C*).

During glucose tolerance test, blood glucose was measured every 30 min for 2 h following a glucose bolus. Male *Exoc5*-SMKO mice showed a slower clearance of glucose during GTT compared with male control mice, and area under the curve of GTT was significantly increased in male *Exoc5*-SMKO mice compared with controls (Fig. 3, *D* and *F*), suggesting impaired glucose tolerance in male *Exoc5*-SMKO mice. However, glucose tolerance and area under the curve of GTT were not significantly different in female *Exoc5*-SMKO mice compared with controls (Fig. 3, *E* and *F*).

We performed insulin tolerance test (ITT) in *Exoc5*-SMKO mice. The mice were fasted for 6 h and given an IP injection of insulin. Blood glucose was measured every 30 min for 2 h. Glucose clearance during ITT was similar between male and female *Exoc5*-SMKO mice and controls (Fig. 3, *G* and *H*). Area under the curve calculations depict a similar response to insulin in both male and female *Exoc5*-SMKO compared with controls (Fig. 3*I*). We also assessed insulin signaling pathway activation in *Exoc5*-SMKO mice compared with controls and found that the insulin signaling pathway component Akt1 is phosphorylated in both *Exoc5*-SMKO and control mice following insulin injection (Fig. 3*J*). Taken together, skeletal muscle knockout of *Exoc5* resulted in increased fasting glycemia and impaired glucose tolerance, but did not impair a functional insulin signaling pathway activation. Of note, gender-specific differences in glucose tolerance were observed in these mice.

### Male *Exoc5*-SMKO mice develop insulin resistance in skeletal muscle

**Figure 4:**
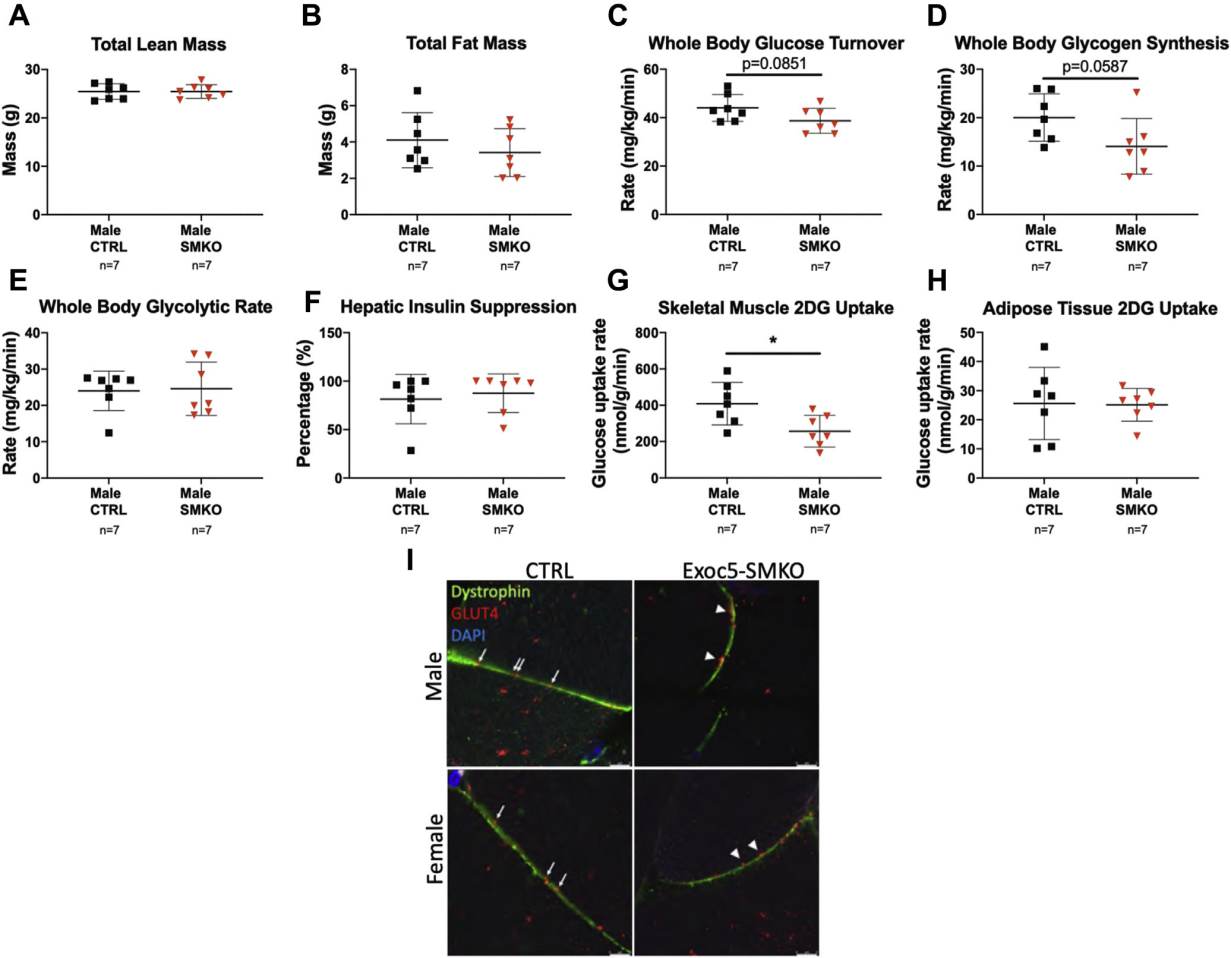
***Exoc5*-SMKO male mice have reduced skeletal muscle glucose uptake and whole-body glucose turnover during hyperinsulinemic–euglycemic clamp.***A* and *B*, proton magnetic resonance spectroscopy was applied to quantify whole-body lean and fat mass in male *Exoc5*-SMKO and control animals. *C*–*F*, hyperinsulinemic–euglycemic clamp was performed in awake mice to assess whole-body glucose turnover (*C*), whole-body glycogen synthesis (*D)*, whole-body glycolysis (*E)*, hepatic insulin action (insulin-mediated percent suppression of basal hepatic glucose production) (*F*), in *Exoc5*-SMKO and control males. *G* and *H*, insulin-stimulated glucose uptake in skeletal muscle (gastrocnemius) (*G*) and white adipose tissue (epididymal) (*H*) was determined following a bolus injection of 2-[^14^C] deoxy-glucose during clamp). *I*, immunofluorescent staining of skeletal muscle tissues of insulin-injected animals. GLUT4 vesicles are in *red* and dystrophin is labeled in *green* in Exoc5-SMKO male and female littermate controls. *White scale bars*, 5 μm. *Error bars* = standard deviation, ∗*p* ≤ 0.05.

Body composition analysis showed that *Exoc5*-SMKO and control male mice had no detectable differences in whole-body fat mass and lean mass (Fig. 4, *A* and *B*). We performed hyperinsulinemic–euglycemic clamp in awake mice to measure whole-body insulin sensitivity and glucose metabolism in individual organs. Whole-body glucose turnover during clamps tended to decrease by 10% in male *Exoc5*-SMKO mice compared with male controls, similarly to whole-body glycogen synthesis, although the differences did not reach statistical significance (*p* = 0.0851 and *p* = 0.0587, respectively) (Fig. 4, *C* and *D*). Whole-body glycolysis was not significantly different in male *Exoc5*-SMKO mice compared with male controls (Fig. 4*E*). Basal and clamp hepatic glucose production and hepatic insulin action were not altered in male *Exoc5*-SMKO mice (Fig. 4*F*).

Insulin-stimulated glucose uptake in individual organs was measured using a bolus injection of labeled 2-deoxyglucose during clamps. Skeletal muscle glucose uptake in response to insulin was significantly decreased in *Exoc5*-SMKO mice compared with controls (Fig. 4*G*). The insulin-stimulated glucose uptake in adipose tissue was not altered in *Exoc5*-SMKO mice (Fig. 4*H*). The impaired skeletal-muscle-specific glucose uptake was not due to defective insulin signaling or total GLUT4 expression because no differences were detected in the mRNA abundance of GLUT4, insulin receptor (InsR), insulin receptor substrate 1 (IRS-1), protein-dependent kinase 1(Pdk1), Rho-related GTP-binding protein (RhoQ), and RAC-beta serine threonine-protein kinase (Akt2) (Fig. 3*J,* Fig. S3). In support of decreased skeletal-muscle-specific glucose uptake, immunofluorescent staining of skeletal muscle tissues of insulin-injected animals revealed a disrupted GLUT4 membrane integration with membrane-proximal accumulation of GLUT4 vesicles in Exoc5-SMKO male and female mice compared with littermate controls (Fig. 4*I*) These results indicate that impaired glucose tolerance and insulin resistance in male *Exoc5*-SMKO mice were mostly due to decreases in insulin-stimulated glucose uptake in skeletal muscle. Based on these results, and our metabolic cage data demonstrating decreased glucose utilization, we expected a switch toward increased fatty acid metabolism in skeletal muscle tissues of our *Exoc5*-SMKO mice. Therefore, we quantified expression of proteins responsible for fatty acid flux as well as mitochondrial content in soleus muscles. Of note, we were not able to detect differences in fatty acid transporter/CD36, lipoprotein lipase (LPL), or mitochondrial components translocase of the outer mitochondrial membrane 20 (TOMM20), voltage-dependent anion-selective channel protein (VDAC1), and cytochrome c oxidase complex four (CoxIV) between groups (Fig. S4).

**Figure 5:**
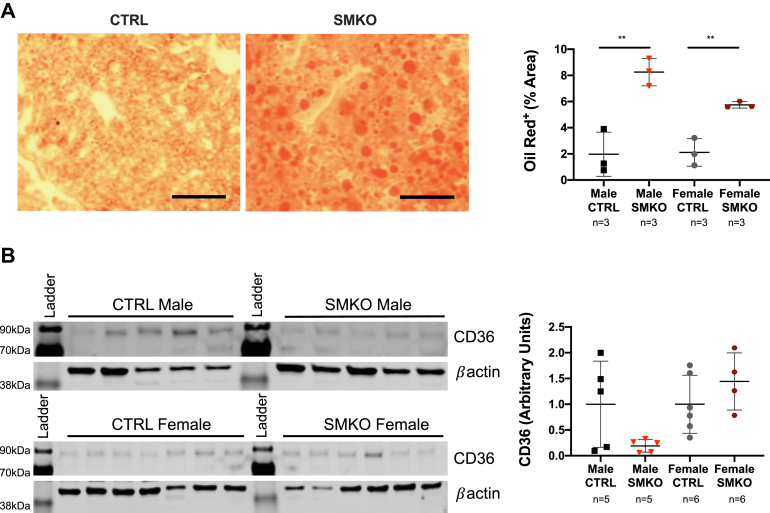
***Exoc5*-SMKO mice had increased hepatic lipid accumulation, yet no difference in insulin-sensitive fatty acid transporter CD36 protein levels was detected.***A*, representative images of control and *Exoc5*-SMKO liver samples stained with Oil Red O (*left panels*). Quantification of the ratio of Oil Red O-stained areas in liver tissues (*right panel*). *B*, images of Western blot analysis (*left panel*) and quantification (*right panel*) of CD36. CD36 levels in *B* as well as LPL and FASN levels in Fig. S5 were sequentially analyzed on a single Western blot, and their respective signal intensities were normalized using the same *β*actin loading control measurements. Thus we show the same *β*actin loading control images in Fig. S5,*B* and *D* as well. *Black scale bars*, 100 μm, *Error bars* = standard deviation, ∗∗*p* ≤ 0.01.

With reduced muscle glucose uptake but no differences in adiposity, we hypothesized a rerouting of increased glucose to the liver. Thus, we evaluated liver morphology and lipid content using Oil Red O staining. We have detected a significantly increased hepatic lipid accumulation in *Exoc5*-SMKO males and females compared with littermate controls (Fig. 5*A*). A significantly higher hepatic triglyceride content was indeed measured in the homogenate of *Exoc5*-SMKO male mice (Fig. S5*A*). We quantified the protein levels of CD36, LPL in the livers of *Exoc5*-SMKO and control mice to decipher if lipid accumulation was due to increased uptake (Fig. 4*B*, and Fig. S5*B*), but found no significant differences between groups. In support of these findings, we detected no differences in liver-specific uptake of the fluorescent lipid analog, BODIPY-palmitate in male or females *Exoc5*-SMKO mice compared with controls (Fig. S5*C*). To investigate if hepatic lipid accumulation was due to *de novo* lipogenesis, we measured protein levels of Fatty Acid Synthase (FASN), as well as mRNA levels of ChREBP, SREBP1, and Acetyl CoA Carboxylase 1 (ACACA), but detected no significant differences (Fig. S5, *D*–*G*). Moreover, there were no differences in serum triglycerides and markers of liver function Aspartate Aminotransferase (AST) and Alanine Aminotransferase (ALT) activity levels (Fig. S5, *H*–*J*).

### Chronic high-fat feeding exacerbates insulin resistance in male *Exoc5*-SMKO mice

**Figure 6:**
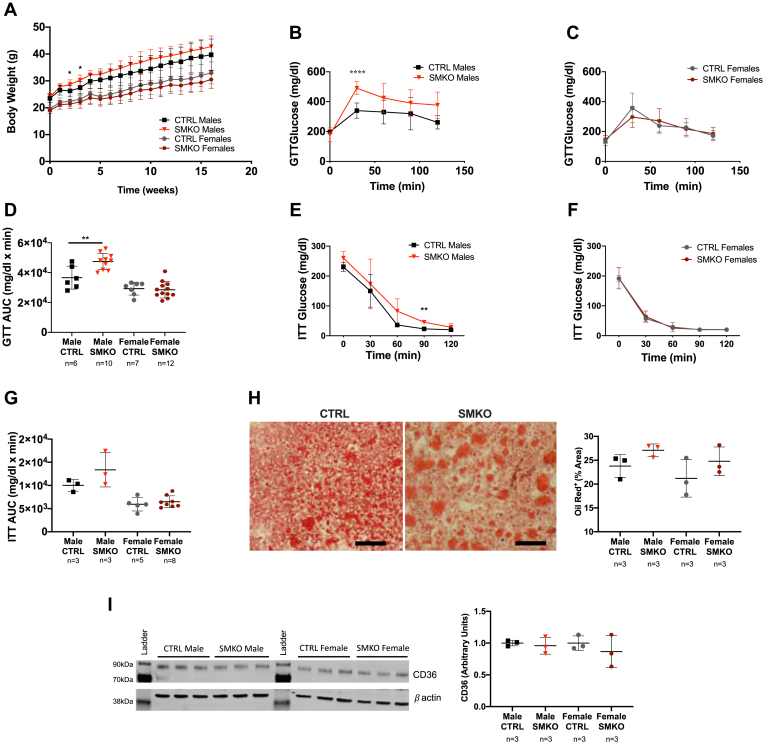
**High-fat diet challenge of skeletal-muscle-specific *Exoc5*-SMKO animals confirms disrupted glucose homeostasis.***A*, weekly body weight measurements of *Exoc5*-SMKO and control animals during a 16-week high-fat diet challenge. *B* and *C*, during the glucose tolerance testing, blood glucose concentrations were measured at 30-min intervals following an intraperitoneal bolus of glucose (1 mg/kg). *D*, quantification of area under the curve for data shown in *B* and *C*. *E* and *F*, during insulin tolerance testing, blood glucose was measured every 30 min for 2 h blood glucose measurement following an intraperitoneal injection of insulin (1 U/kg). *G*, area under the curve calculations for data presented in *E* and *F*. *H*, representative images of control and *Exoc5*-SMKO liver samples stained with Oil Red O (*left panel*) and quantification of the ratio of Oil Red O-stained areas in liver tissues (*right panel*) in *Exoc5*-SMKO and littermate control animals following. *Black scale bars*, 100 μm. Images of Western blot analysis (*upper panel*) used for the quantification of *I*, images of Western blot analysis (*left panel*) used for the quantification of CD36 protein levels (normalized to *β*-actin) in the livers of *Exoc5*-SMKO and control mice (*right panel*). *Error bars* = standard deviation, ∗*p* ≤ 0.05; ∗∗*p* ≤ 0.01, ∗∗∗∗*p* ≤ 0.0001.

Male and female *Exoc5*-SMKO and control mice were challenged with a high-fat diet (HFD) for 16 weeks to assess if impaired exocyst function affects diet-induced changes in energy metabolism. Body weights were measured weekly, and after 16 weeks of HFD, we did not detect significant differences in body weights between knockout and control animals (Fig. 6*A*). GTT revealed significantly reduced glucose tolerance in HFD-fed male *Exoc5*-SMKO mice compared with controls, whereas no difference in glucose tolerance could be detected in HFD-fed *Exoc5*-SMKO females compared with the littermate controls (Fig. 6, *B*–*D*).

ITT of the HFD-fed cohorts also showed reduced glucose clearance in male *Exoc5*-SMKO mice compared with controls, but no differences between female *Exoc5*-SMKO mice and controls after HFD (Fig. 6, *E*–*G*). While fasting serum insulin levels increased compared with regular diet-fed cohort, there were no significant differences in fasting serum insulin and islet size between high-fat diet-fed *Exoc5*-SMKO mice and littermate controls (Fig. S6, *A* and *B*). There were no differences in fasting serum triglyceride levels either (Fig. S6*C*). Of note, when we evaluated hepatic lipid accumulation in the HFD-fed animals, we could not detect a significant difference between *Exoc5*-SMKO and control animals in either sex (Fig. 6*H*). In addition, we did not detect significant differences in CD36 (Fig. 6*I*), LPL, and FASN (Fig. S6, *D* and *E*) protein levels in the livers of *Exoc5*-SMKO mice either.

## Discussion

Insulin-stimulated glucose uptake by skeletal muscle is an essential part of glucose homeostasis (18). Insulin stimulation in a healthy individual results in the exocytosis of the GLUT4 and subsequent glucose uptake into major metabolic tissues such as skeletal muscle, fat, and heart (19). Discordance in the mechanisms that coordinate the GLUT4 exocytosis in skeletal muscle may contribute to the primary dysfunction in T2D (20).

Previous studies using gene knockout animals have demonstrated that GLUT4 and its function are essential for maintaining glucose homeostasis. The heterozygous whole-body knockout of GLUT4 in mice resulted in decreased glucose uptake in fat and muscle cells, increased adipocyte size, hypertension, and cardiac hypertrophy, as well as hepatic steatosis (21). Furthermore, knockout of GLUT4 in skeletal muscle alone resulted in reduced glucose tolerance, and the decreased serum lactate and *β*-hydroxybutyrate levels in skeletal muscle GLUT4-null mice indicated decreased glucose oxidation (22). However, GLUT4 overexpression in adipose tissue was able to rescue insulin resistance in mice lacking GLUT4 in skeletal muscle (23).

The eight-protein exocyst complex is involved in vesicle docking and has been evolutionarily conserved from yeast to humans (24). It has been extensively demonstrated that the exocyst complex is essential for insulin-stimulated GLUT4 exocytosis in cultured adipocytes (11, 12, 25). The siRNA knockdown of EXOC6/6b inhibited GLUT4 exocytosis in 3T3-L1 adipocytes (10). On the other hand, overexpression of EXOC3 and EXOC4 in 3T3-L1 adipocytes increased the magnitude of glucose transport in response to insulin (11). There have been very few studies directly examining the role of the exocyst complex in skeletal muscle tissues. We have previously demonstrated that chemical inhibition of the exocyst complex and the heterozygous knockout of *Exoc5* lead to a reduction in insulin-stimulated GLUT4 membrane delivery and glucose uptake in rat skeletal myoblasts (17).

Here, we are the first to create an *in vivo* model of a skeletal-muscle-specific knockout of a component of the exocyst complex. *Exoc5*-SMKO mice appeared to have normal neuromuscular function as well as an intact insulin signaling pathway activation, and no differences in the expression levels of genes that are involved in insulin-stimulated glucose uptake were detected.

Food and water intake, activity level, body weight, and body composition were similar between *Exoc5*-SMKO and control animals as measured using metabolic cages and proton magnetic resonance spectroscopy. Our indirect calorimetry data suggest that the skeletal muscle depletion of EXOC5 results in a metabolic shift from glucose utilization toward increased amino acid and fatty acid metabolism in both sexes. A reduced carbohydrate utilization as an energy substrate is likely due to an impaired glucose uptake in skeletal muscle. In support of our findings, insulin-resistant mice were previously shown to have RER of approximately 0.8 and an overnight fasting glucose of 600 mg/dl starting at 15 weeks of age, while wild-type mice had an RER of 0.9 and a fasting glucose level around 100 mg/dl (26).

Hyperinsulinemic–euglycemic clamp demonstrated that insulin sensitivity was reduced selectively in skeletal muscle of male *Exoc5*-SMKO mice. Glucose uptake in adipose tissue was similar between *Exoc5*-SMKO mice and controls. No differences in whole-body glycogen synthesis were detected in *Exoc5*-SMKO mice. Although, 70 to 90% of glucose absorbed by skeletal muscle is converted into glycogen during a hyperinsulinemic–euglycemic clamp (27) and skeletal muscle stores 80% of the of the body’s glycogen (27), the effect of the impaired skeletal muscle glucose uptake was insufficient to alter these parameters. Overnight fasting glucose is higher in *Exoc5*-SMKO mice, and our findings indicate that the excess glucose may be routed to the liver, as suggested by hepatic lipid accumulation. Yet, we were not able to detect any significant differences in hepatic lipid uptake or *de novo* lipogenesis and further investigation is warranted. The hyperinsulinemic–euglycemic clamp data also showed no effects on hepatic insulin sensitivity as insulin maximally suppressed hepatic glucose production in *Exoc5*-SMKO and control mice. It should be noted, however, that since hepatic glucose production was maximally suppressed by insulin during clamp, potential effects on hepatic insulin action may have been masked in this study.

While body weights and body composition of *Exoc5*-SMKO and littermate control mice were similar for both sexes, glucose tolerance was significantly reduced in male *Exoc5*-SMKO mice only. The glucose tolerance of female *Exoc5*-SMKO mice was not altered. Differences in the glucose responsive tissues between male and female mice may protect female mice from dysregulation of glucose homeostasis. Studies on mice have shown that there are more than 300 differentially expressed genes in skeletal muscle between males and females (28). Thus, the gender-specific effects we describe in the glucose homeostasis of our *Exoc5*-SMKO animals warrant further investigation.

No differences in insulin tolerance were observed between *Exoc5*-SMKO and control mice. It has been documented that insulin tolerance test may lack the sensitivity necessary to resolve metabolic differences in mice, in particular when glucose metabolism is altered in selective metabolic organs (29). Male *Exoc5*-SMKO had lower insulin levels yet we did not find any significant differences in pancreatic islet size or insulin-positive cell ratios compared with controls.

The *ad libitum* HFD treatment closely mimics the effect of the nutrient-dense Western diet in humans. After 16 to 20 weeks, mice gain 20 to 30% more weight than mice fed normal chow and hyperglycemia usually begins after 4 weeks (30). After a 16-week HFD, the body weights of *Exoc5*-SMKO and littermate controls were similar. Male *Exoc5*-SMKO mice challenged with a HFD for 16 weeks had decreased glucose tolerance and reduced glucose clearance in response to exogenous insulin, and female *Exoc5*-SMKO mice were similar to controls. This may be due to estrogen’s role in female mice contributing to an increased reliance on lipid oxidation (31), masking any possible effects of altered skeletal muscle glucose uptake. The differences in male and female muscle and adipose tissue anatomy and physiology were mentioned earlier.

Lipid dysregulation, especially elevated serum triglycerides are commonly associated with T2D (32). Elevated serum triglycerides can be a potential sign of liver dysfunction (32). However, fasting serum insulin and serum triglycerides were not significantly different between *Exoc5*-SMKO and control mice challenged with a HFD. Of note, no significant impairment of liver function could be detected in our *Exoc5*-SMKO mice compared with controls.

Our previous study demonstrated that reducing the amount of EXOC5 impairs the ability of mouse skeletal muscle cells to uptake glucose in response to insulin (17). This was due to a failure to increase the amount of GLUT4 transporters at the plasma membrane. In this study, we highlight the central role of the exocyst complex in insulin-stimulated glucose uptake in skeletal muscle *in vivo*. We demonstrate that skeletal-muscle-specific knockout of *Exoc5* causes insulin resistance in mice, and impaired skeletal-muscle-specific glucose uptake triggers a shift from carbohydrate metabolism toward lipid utilization and increases hepatic lipid accumulation as a possible substrate redistribution from skeletal muscle tissues to the liver.

Ongoing work will further investigate the molecular mechanism of exocyst-mediated GLUT4 trafficking in skeletal muscle.

## Experimental procedures

### Animals

The “floxed” Exoc5 (Exoc5^fl/fl^) mouse strain (kindly provided by Dr Ben Fogelgren, University of Hawaii) was generated and validated as previously described (33). To inactivate *Exoc5* in a muscle-specific manner, we obtained HSA-MCM mice (Tg(ACTA1-cre/Esr1∗)2Kesr/J) from Dr Karyn Esser from the University of Kentucky Center for Muscle Biology. This HSA-MCM strain has a tamoxifen-inducible form of Cre recombinase (CreERT2) under the control of the human skeletal actin (HSA) promoter (34). The B6.Cg-Gt(ROSA)26Sortm9(CAG-tdTomato) Hze/J reporter mouse strain was used to detect Cre recombinase activity through Cre-activated expression of the tdTomato red fluorescent protein (kindly provided by Dr Michelle Tallquist, University of Hawaii) (35).

All animal procedures and protocols were carried out in accordance with IACUC specifications approved by the University of Hawaii Animal and Veterinary Services and the University of Massachusetts Medical School.

### Tamoxifen treatment

Mice at 6 to 8 weeks of age were given tamoxifen-containing chow (Envigo cat #TD. 130860) *ad libitum* for 2 weeks to induce skeletal-muscle-specific Cre recombinase activation and subsequent gene recombination. To assess tissue-specific Cre recombinase activation, heterozygous Exoc5^fl/+^; HSA-MCM/+;tdTomato/+ mice, age- and sex-matched littermate control animals of Exoc5^fl/fl^; +/+,tdTomato/+ genotype (both carrying the tdTomato reporter allele) were subjected to tamoxifen treatment. The Cre activation was assessed based on expression of the red fluorescent tdTomato in various tissues. To evaluate glucose homeostasis, Exoc5^fl/fl^;HSA-MCM/+ (*Exoc5*-SMKO) mice and age- and sex-matched littermate animals of Exoc5^fl/fl^; +/+ (control) genotype were used in all experiments.

### High-fat diet and body composition analysis

Ten- to twelve-week-old *Exoc5*-SMKO and control mice were given a HFD (Envigo cat #TD.07011; containing 29% Fat, 30% Carbohydrate, 26% protein by weight) for 16 weeks. Body weight was measured every 7 days. Whole-body fat and lean mass of male 20-week-old male *Exoc5*-SMKO and control mice were noninvasively assessed using Proton magnetic resonance spectroscopy (^1^H-MRS, EchoMRI, EchoMedical Systems) (36).

### Motor function testing

With assistance from the JABSOM Murine Behavior and Metabolic Research Support Facility (http://mbmc.jabsom.hawaii.edu), open-field and Rotarod tests were performed to assess locomotion and motor coordination as previously described (37).

### Glucose and insulin tolerance test

For intraperitoneal glucose tolerance test (GTT), overnight fasted mice were injected intraperitoneally (IP) with glucose (1 mg/kg), and blood glucose levels were measured using a OneTouch Ultra handheld glucometer. For insulin tolerance test, mice were fated for 6 h, insulin was subsequently delivered *via* intraperitoneal (IP) injection at 0.75 IU/kg, and glycemia was measured using a OneTouch Ultra handheld glucometer.

### Measurement of energy balance and indirect calorimetry

With assistance from the University of Hawaii Murine Behavior and Metabolic Research Support Facility (http://mbmc.jabsom.hawaii.edu), whole-body energy balance was assessed *via* indirect calorimetry using the Panlab Oxylet*Pro* System (Harvard Apparatus, Barcelona, Spain). Adult (16-week-old) male *Exoc5*-SMKO and control animals were weighed and individually housed in metabolic cages. Following a 24-h acclimation period, food and water intake, physical activity, and energy expenditure (O_2_ consumption, CO_2_ production) were measured over a 24-h period. Data collection and calculations were performed using the Panlab METABOLISM software.

### Hyperinsulinemic–euglycemic clamp to assess insulin sensitivity *in vivo*

Hyperinsulinemic–euglycemic clamps were conducted in male 20-week-old *Exoc5*-SMKO and control mice as previously described (38). At the end of the clamps, mice were anesthetized, and tissues were taken for biochemical analysis.

Glucose concentrations during clamps were analyzed using 10 μl plasma by a glucose oxidase method on Analox GM9 Analyser (Analox Instruments Ltd). Plasma concentrations of [3-^3^H]glucose, 2-[^14^C]DG, and ^3^H_2_O were determined following deproteinization of plasma samples as previously described (29). For the determination of tissue 2-[^14^C]DG-6-Phosphate content, tissue samples were homogenized, and the supernatants were subjected to an ion-exchange column to separate 2-[^14^C]DG-6-P from 2-[^14^C]DG.

Rates of basal hepatic glucose production (HGP) and insulin-stimulated whole-body glucose turnover were determined as previously described (38).

### Serum and hepatic triglyceride, serum insulin, and HbA1c analysis

Male and female *Exoc5*-SMKO and control mice were fasted for 6 h and upon euthanasia, blood was drawn by cardiac puncture. Plasma was separated using BD Microtainer Capillary Blood Collector and BD Microgard Closure tubes and stored at −20 °C. Serum triglyceride was measured using a Triglyceride Colorimetric Assay Kit (Cayman Chemical Catalog # 10010303) per the manufacturer’s recommendation with a SpectraMax M3 microplate reader (Molecular Devices, LLC). Serum insulin was measured using a Mouse Ultra Sensitive Insulin ELISA kit (ALPCO catalog #80-INSMSU-E01).

### Histology and Immunofluorescence

All samples were imaged using an Olympus BX41 microscope. Image processing, quantification of pancreatic islet areas, and oil red staining were performed using ImageJ (39).

For immunofluorescence, deparaffinized and rehydrated sections were subjected to a citrate-based antigen retrieval. After permeabilization and blocking, sections were incubated with primary antibodies (anti-insulin (Cat #66198-1-Ig, Proteintech Group, Inc); anti-glucagon (Cat # 15954-1-AP,Proteintech Group, Inc)) overnight at 4 °C and incubated with appropriate fluorescent secondary antibodies (DyLight 488 Anti-Rabbit IgG and DyLight 594 Anti-Mouse, Vector Laboratories) for detection. Cell nuclei were stained with DAPI.

In the sections stained for insulin and glucagon, the borders of each pancreatic islet were identified morphologically and marked using the pen tool in ImageJ. The area of each islet was calculated with ImageJ software using the set scale function corresponding to the 20x scale bar. Insulin-positive nuclei were counted based on fluorescent signal. All islets with an area >566 μmˆ2 (corresponding to a diameter of >27 μm), approximately corresponding to islets containing a minimum of eight nuclei, were regarded for analysis.

### Western blot analysis

Skeletal muscle proteins were extracted using an extraction buffer (20 mM Tris, 137 mM NaCl, 2.7 mM KCl, 1 mM MgCl2, 1% Triton X-100, 10% glycerol, 1 mM EDTA, 1 mM DTT) and a mechanical homogenizer. Lysates were separated on a gradient (4–15%) SDS-PAGE gel, and proteins were transferred onto a polyvinylidene fluoride membrane. The membrane was incubated with primary antibodies (AKT1, Santa Cruz, sc-5298; EXOC5, Proteintech, 17593-1-AP; GAPDH, Proteintech, 60004-1-Ig; FASN, Proteintech, 10624-2-AP; CD36, Novus, NB400-144; LPL, Novus, AF7197; pAKT (Ser 473), Cell Signaling, 4060T; TOMM20, Abcam, ab186735; VDAC1, Abcam, ab129002; Vinculin, Abcam, ab129002; CoxIV, Cell Signaling, 4850) diluted 1:1000 in 5% BSA in PBST at 4 °C overnight, and with fluorescent secondary antibodies (IRDye 800 conjugated goat anti-rabbit IgG, LI-COR Biosciences, 926-32211; IRDye 680RD donkey anti-mouse IgG, LI-COR Biosciences, 926-68072) diluted 1:15,000 in 5% BSA in PBST with 0.02% SDS. Blots were scanned with a LI-COR Odyssey Clx scanner, and densitometry was performed using the Image Studio software (LI-COR Biosciences).

### Quantitative real-time PCR analysis

Total RNA from the quadriceps femoris muscle was collected using the RNeasy Fibrous Tissue Mini Kit (Qiagen Germany). The iScript reverse transcriptase (Bio-Rad) was used to generate cDNA from extracted RNA samples. Quantitative PCR was performed on a CFX96 Real Time System (Bio-Rad), using the iTaq Universal SYBR Green Supermix (Bio-Rad) using predesigned primer assays (PrimeTime, Integrated DNA Technologies). Gene expression was normalized to Rplp0. Quantitative PCR primer sequences are listed in Table S1.

### BODIPY—palmitate uptake assay

*In vivo* hepatic uptake of lipid was measured by fasting *Exoc5*-SMKO and littermate controls overnight. A single IP injection of 50 μg BODIPY-conjugated palmitate diluted in 1% BSA was administered. Mice were sacrificed by isoflurane overdose 1 h after injection and tissues were harvested immediately. Livers were mechanically homogenized following the addition of 2 ml of radioimmunoprecipitation assay buffer. Lysates were centrifuged at 1000*g* and the fluorescence of the supernatant was measured ex/em 488/512. The fluorescence values were normalized by protein concentration determined by bicinchoninic acid assay.

### Statistical methods

Graphs show mean ± standard deviation, unless otherwise indicated. Statistical comparisons between the subgroups were assessed by one-way analysis of variance (ANOVA). In all other cases, a Student’s *t*-test was performed to calculate *p* values and determine if there was a significant difference between two groups. *p* values of less than 0.05 were considered statistically significant. Area under the curve (positive incremental) values were calculated based on the trapezoid model, relative to the baseline, using the GraphPad Prism software (GraphPad, Inc) (40).

## Data availability

All relevant data generated during this study are included in this published article (and its supplementary information files).

## Supporting information

This article contains supporting information.

## Conflict of interest

The authors disclose of no conflict of interest.
