# Supplementary material for: Disrupted glucose homeostasis and skeletal-muscle-specific glucose uptake in an exocyst knockout mouse model
Source: J Biol Chem. 2021 Feb 27;296:100482. doi: 10.1016/j.jbc.2021.100482 (PMC8027262; doi:10.1016/j.jbc.2021.100482)
Supplement: Supplemental Figures S1–S6 and Table S1 [file mmc1.pdf]

SUPPORTING INFORMATION

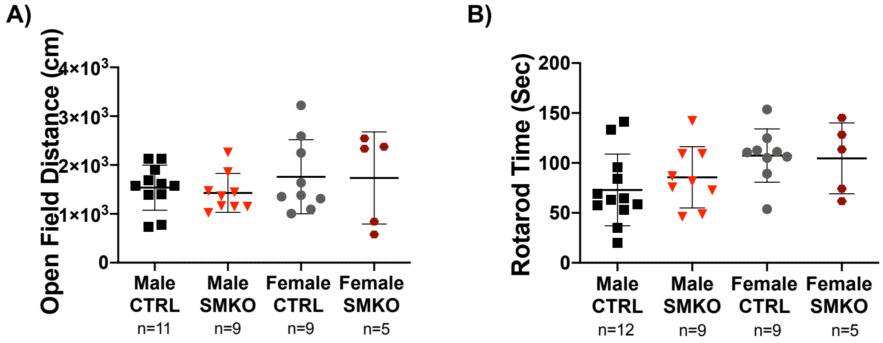

Supporting Information Figure 1. No differences in neuromuscular function were detected in *Exoc5*-SMKO mice. A) Locomotive activity was evaluated using 5-minute trials of open field tests. **B)** Eight five-minute trials of rotarod motor performance tests were performed with accelerating speed over 2 days. Error bars= standard deviation.

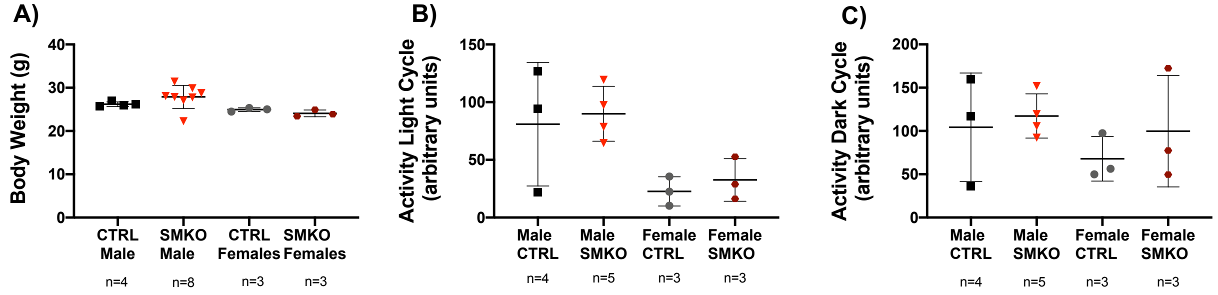

Supporting Information Figure 2. No differences in body weight and activity level were detected during metabolic cage testing in *Exoc5*-SMKO mice. A) body weights were measure prior to metabolic cage testing. **B and C)** Activity levels were measured for 24 hours and specified between light and dark periods. Error bars= standard deviation

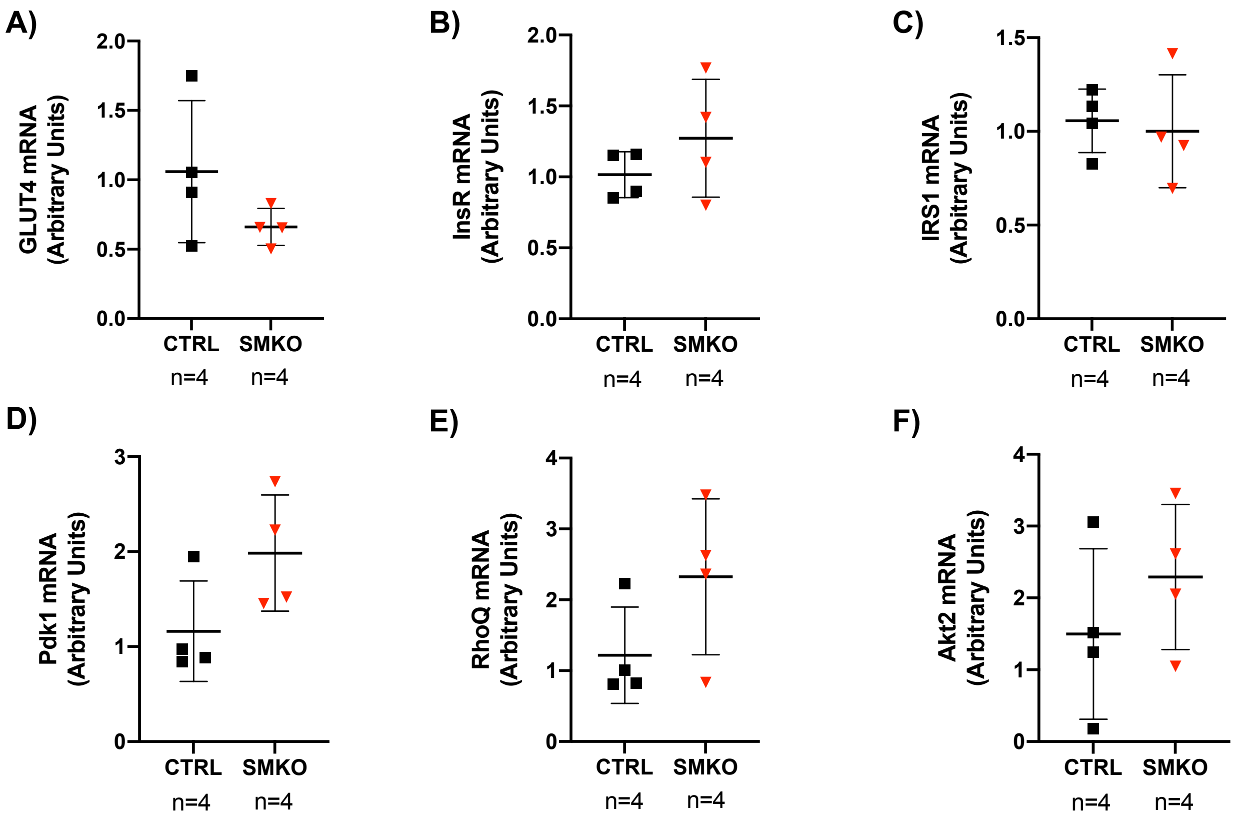

Supporting Information Figure 3. Gene expression analysis of skeletal muscle demonstrate no differences in insulin signaling pathway components. A-G) Real-time quantitative PCR was used to measure mRNA abundance of insulin signaling pathway components in the quadriceps femoris of *Exoc5*-SMKO and CTRL mice. Error bars= standard deviation.

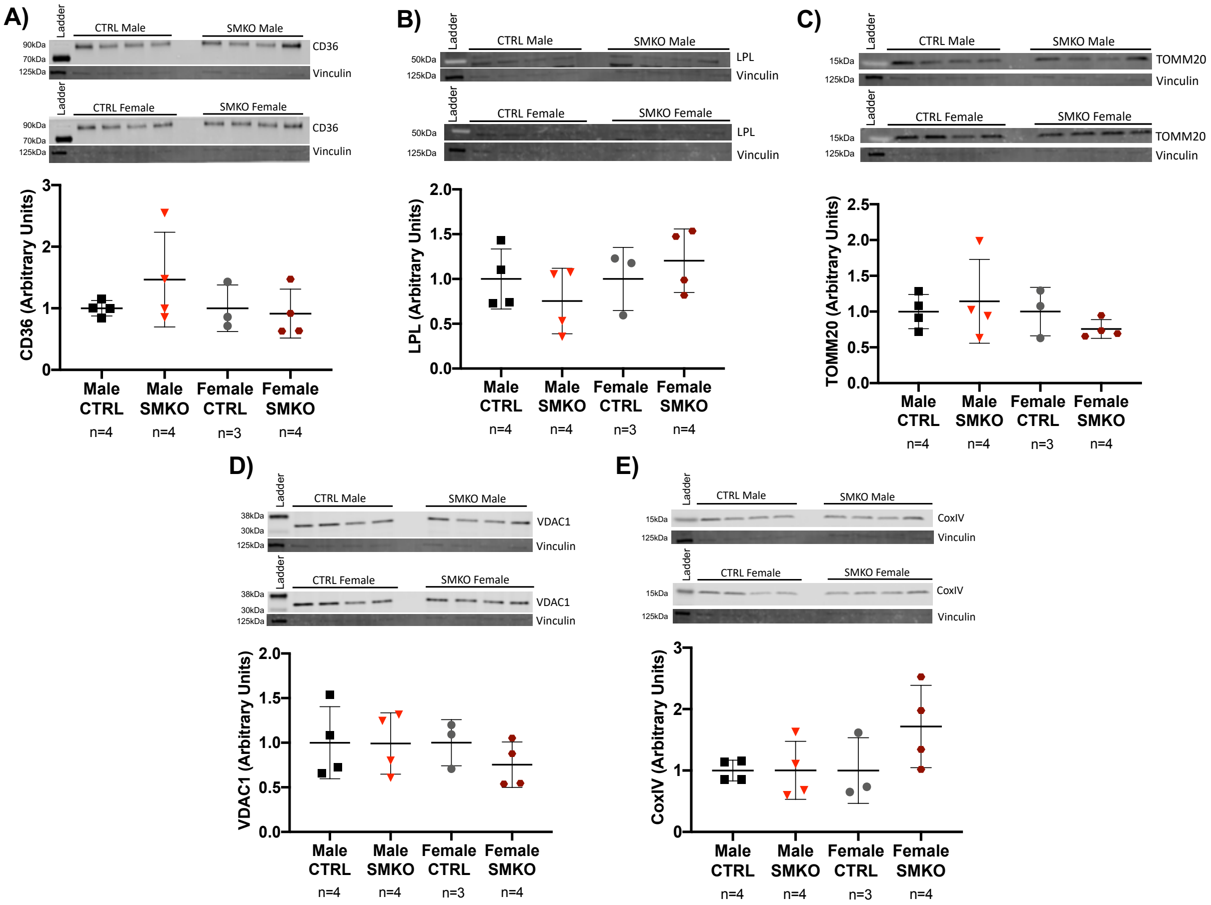

Supporting Information Figure 4. No significant differences in fatty acid metabolism and mitochondrial genes were found in the soleus of skeletal muscle specific EXOC5 knock out mice. Images of Western blot analysis (upper panel) used for the quantification of **A)** CD36, **B)** LPL, **C)** TOMM20, **D)** VDAC1, **F)** CoxIV protein levels (normalized to Vinculin) in the livers of *Exoc5*-SMKO and control mice (lower panel). CD36, TOMM20, and VDAC1 levels were sequentially analyzed on a single Western blot, and their respective signal intensities were normalized using the same vinculin loading control measurements. Thus we show the same vinculin loading control images in A), C) and D). LPL and CoxIV were analyzed in in a similar manner, thus we include the same vinculin loading control images in B) and E).Error bars= standard deviation.

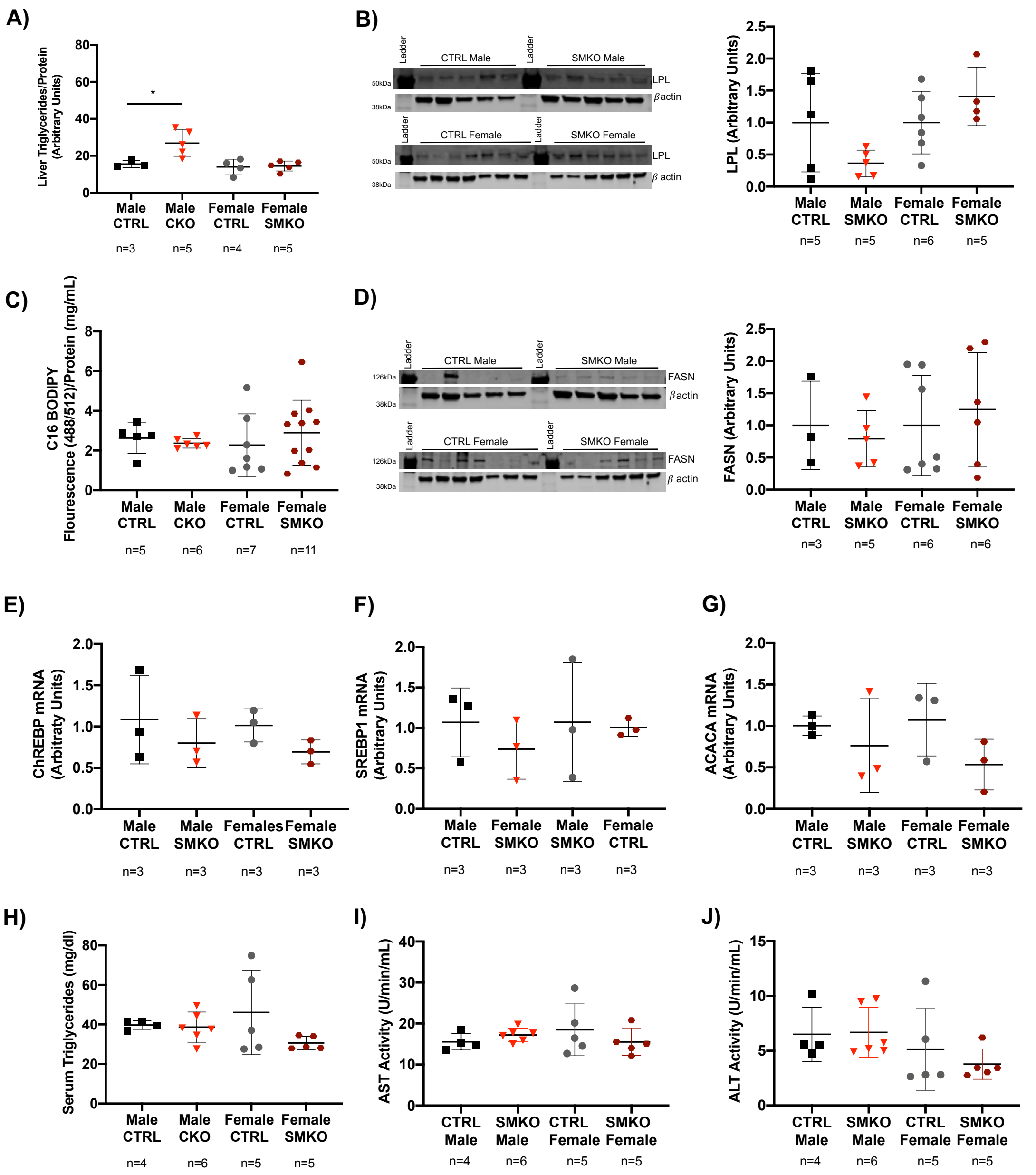

Supporting Information Figure 5. Although higher triglyceride levels were detected in the livers of *Exoc5*-SMKO, no differences in fatty acid metabolism genes were detected in the livers of *Exoc5*-SMKO mice, as well as differences in serum ALT, AST, and triglycerides. A) Livers were homogenized, and triglyceride content was measured. Images of Western blot analysis (upper panel) used for the quantification of **B)** LPL protein levels (normalized to βactin) in the livers of *Exoc5*-SMKO and control mice. **C**) Hepatic uptake of a fluorescent lipid analogue C16-BIPY was measured in fasted mice. **D)** Western analysis of FASN protein levels (normalized to βactin) in the livers of *Exoc5*-SMKO and control mice. **E-G)** Real-time quantitative PCR was used to measure mRNA abundance of ChREBP, SREBP, and ACACA in the livers of *Exoc5*-SMKO and CTRL mice. Serum triglyceride concentration (**H**), aspartate aminotransferase (AST) (**I**) and alanine aminotransferase (ALT) (**J**) activity levels were measured. LPL and FASN levels (as well as CD36 levels in Figure 5) were sequentially analyzed on a single Western blot, and their respective signal intensities were normalized using the same β actin loading control measurements. Thus we show the same β actin loading control images in B) and D) and Figure 5B. Error bars= standard deviation, *p≤0.05.

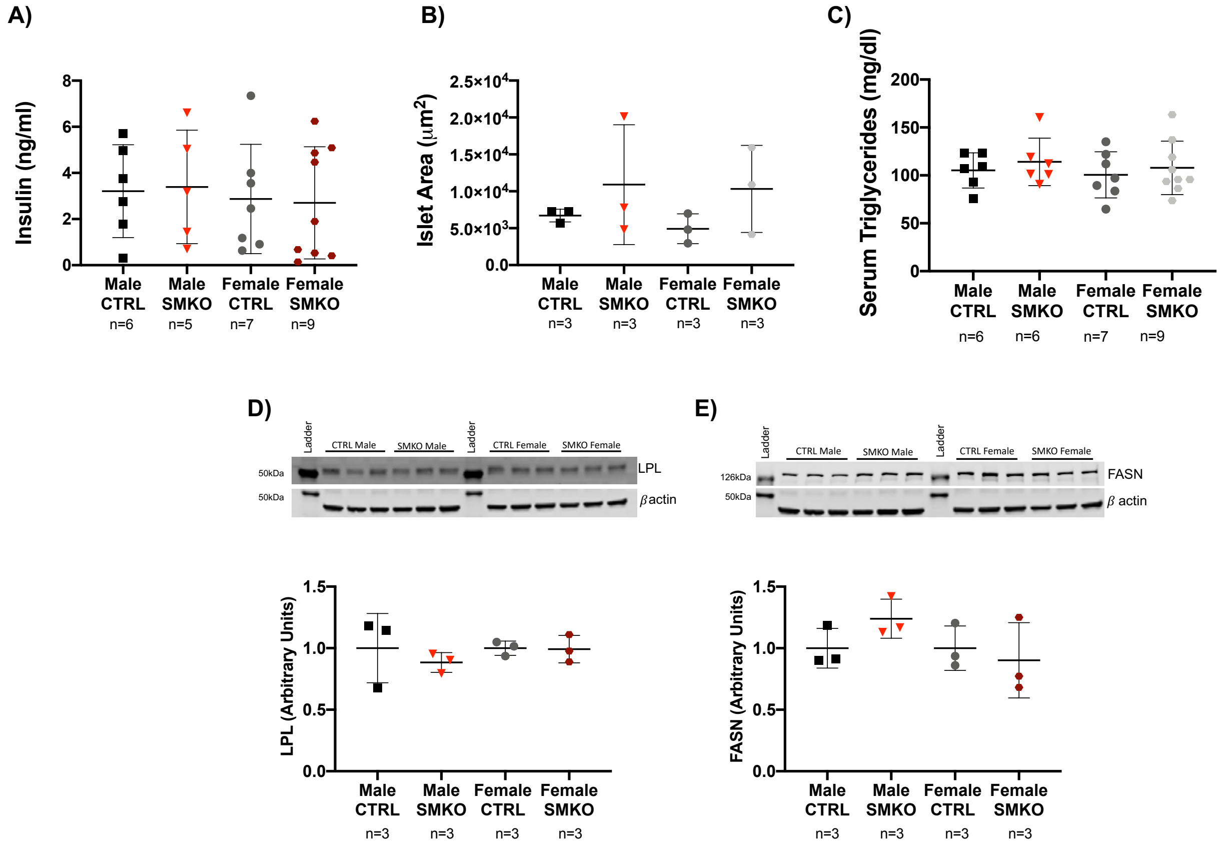

Supporting Information Figure 6. No differences in fasting serum insulin and triglycerides, as well as liver expression of LPL and FASN of HFD challenged CTRL and *Exoc5*-SMKO mice. A) Fasting insulin levels were measured using an ELISA assay. **B)** Histological methods were used to measure pancreatic islet areas**. C)** Serum triglyceride levels were measured. **D)** LPL, and **E)** FASN protein levels (normalized to β actin) in the livers of *Exoc5*-SMKO and control mice. LPL and FASN levels were sequentially analyzed on a single Western blot, and their respective signal intensities were normalized using the same β actin loading control measurements. Thus we show the same β actin loading control images in E) and F). Error bars= standard deviation.

Mm.PT.58.12492865	Acaca	Primer 1: 5'-AACATCCCCACGCTAAACAG-3'<br>Primer 2: 5'-GTCCAACAGAACATCGCTGA-3'
Mm.PT.58.8836469	Akt2	Primer 1: 5'-ACAAGCCAAAGTCAGTGATCT-3'<br>Primer 2: 5'-GAGATTGTGTCAGCTCTGGAG-3'
Mm.PT.56a.33592172	ChREBP	Primer 1: 5'-CACCTCTTCGAGTGCTTGAG-3'<br>Primer 2: 5'-TTGTTCAGCCGGATCTTGTC-3'
Mm.PT.58.32744074	Exoc4	Primer 1: 5'-ATGAGGATACAGGAGATGAGGT-3'<br>Primer 2: 5'-ATGTGGAGAGTATGGATTACGAC-3'
Mm.PT.58.10976129	Exoc5	Primer 1: 5'-GCTGGCATTCTAAGTCATGGTA-3'<br>Primer 2: 5'-AGATAAAGGAAGCAGCAGACG-3'
Mm.PT.58.9275253	Insr	Primer 1: 5'-CAATTCCATCACTACCAGCGT-3'<br>Primer 2: 5'-TCAATGAGTCAGCCAGTCTTC-3'
Mm.PT.58.43919344	Irs1	Primer 1: 5'-TGTGAATTGTGAAATAGTTCGAGTC-3'<br>Primer 2: 5'-CCAGCATCAGCTTCCAGAA-3'
Mm.PT.58.10680444	Pdk1	Primer 1: 5'-GCCTAGCGTTCTCATAGCC-3'<br>Primer 2: 5'-ACCAGCACTCCTTATTGTTCG-3'
Mm.PT.58.5834541	Rhoq	Primer 1: 5'-GATAGCCTCATCAAACACAGTCT-3'<br>Primer 2: 5'-GGAACAAGGACAGAAACTAGCA-3'
Mm.PT.58.43894205	Rplp0	Primer 1: 5'-CGCTTGTACCCATTGATGATG-3'<br>Primer 2: 5'-TTATAACCCTGAAGTGCTCGAC-3'
Mm.PT.58.7590689	Slc2a1	Primer 1: 5'-GTGGTGAGTGTGGTGGATG-3'<br>Primer 2: 5'-AGTTCGGCTATAACACTGGTG-3'
Mm.PT.58.9683859	Slc2a4	Primer 1: 5'-GAGAATACAGCTAGGACCAGTG -3'<br>Primer 2: 5'-TCTTATTGCAGCGCCTGAG-3'
Mm.PT.58.8508227	SREBP1c	Primer 1: 5'-CGAGATGTGCGAACTGGAC-3'<br>Primer 2: 5'-GTCACTGTCTTGGTTGTTGATG-3'

Supporting Information Table 1. qPCR primer sequences
